# Biological and disease hallmarks of Alzheimer’s disease defined by Alzheimer’s disease genes

**DOI:** 10.3389/fnagi.2022.996030

**Published:** 2022-11-09

**Authors:** Shin Murakami, Patricia Lacayo

**Affiliations:** Department of Basic Sciences, College of Osteopathic Medicine, Touro University California, Vallejo, CA, United States

**Keywords:** Alzheimer’s disease, genetics, comorbidity, diabetes, hypertension, cancer, metabolism

## Abstract

An increasing number of genes associated with Alzheimer’s disease (AD genes) have been reported. However, there is a lack of an overview of the genetic relationship between AD and age-related comorbidities, such as hypertension, myocardial infarction, and diabetes, among others. Previously, we used Reactome analysis in conjunction with the AD genes to identify both the biological pathways and the neurological diseases. Here we provide systematic updates on the genetic and disease hallmarks defined by AD genes. The analysis identified 50 pathways (defined as biological hallmarks). Of them, we have successfully compiled them into a total of 11 biological hallmarks, including 6 existing hallmarks and 5 newly updated hallmarks. The AD genes further identified 20 diverse diseases (defined as disease hallmarks), summarized into three major categories: (1) existing hallmarks, including neurological diseases; (2) newly identified hallmarks, including common age-related diseases such as diabetes, hypertension, other cardiovascular diseases, and cancers; (3) and other health conditions; note that cancers reportedly have an inverse relation with AD. We previously suggested that a single gene is associated with multiple neurological diseases, and we are further extending the finding that AD genes are associated with common age-related comorbidities and others. This study indicates that the heterogeneity of Alzheimer’s disease predicts complex clinical presentations in people living with AD. Taken together, the genes define AD as a part of age-related comorbidities with shared biological mechanisms and may raise awareness of a healthy lifestyle as potential prevention and treatment of the comorbidities.

## Introduction

Alzheimer’s disease is the major cause of dementia. According to the Centers for Disease Control and Prevention (CDC), 5.8 million Americans were living with AD in 2020 (Matthews et al., 2018). Pathological characteristics of AD include diffuse and neuritic plaques characterized by amyloid plaques and neurofibrillary tangles (Vaz and Silvestre, 2020; Sherva and Kowall, 2022). Despite these pathological characteristics, the brain pathology and progression of AD are clinically heterogeneous and thus, a clinically complex disease (Ferrari and Sorbi, 2021). Therefore, AD can be classified as late-onset (LOAD), early-onset (EOAD), and autosomal dominant forms of which LOAD is the most frequent.

AD is also highly heritable and genetically heterogeneous (Vahdati Nia et al., 2017; Sherva and Kowall, 2022). Linkage analysis, genome-wide association studies and candidate gene studies have identified Alzheimer’s disease genes (AD genes). Of the 680 AD genes that are reported in the Alzgene database,^1^ 356 genes were found to be associated with AD (Vahdati Nia et al., 2017). Four genes are known to cause AD (APP, PSEN1, and PSEN2) or to be a risk factor (ApoE4). Based on the AD genes, a previous study identified biological Reactome pathways as biological hallmarks (Vahdati Nia et al., 2017). Another important finding was that AD genes are associated with 5 neurological diseases, suggesting a single gene alteration can be associated with multiple forms of neurological diseases. Here we updated and organized the biological hallmarks as well as disease hallmarks. Surprisingly, the results suggest more diverse biological hallmarks and include not only neurological diseases but also common age-related diseases, which we summarize in this study.

## Methods

The method has been described (Vahdati Nia et al., 2017). The AD genes have been validated and described earlier (Bertram et al., 2007; Bateman et al., 2012; Vahdati Nia et al., 2017). We used 356 AD genes. We used the updated Reactome pathway knowledgebase 2022^2^ (Gillespie et al., 2022) and another knowledgebase, GeneAnalytics^3^ (Ben-Ari Fuchs et al., 2016). STRING-DB (Version 11.5) was used to display gene interaction networks^4^ (Szklarczyk et al., 2017). The Reactome pathways were set to a threshold of *p*-value ≤ 1.00E-05. To eliminate redundancies, we categorized the pathways into a spectrum ranging from general to specific: general Reactome pathways (general hallmarks), more specific pathways (more specific hallmarks), and specific pathways (specific hallmarks). For example, the genes involved in the transport of small molecules (e.g., minerals, proteins, lipids and fat-soluble vitamins) provided a Reactome analysis output that further ranks the order from general to specific (D’Eustachio, 2006): Transport of small molecules (as general hallmarks) → Plasma lipoprotein assembly, remodeling, and clearance (as more specific hallmarks) → Plasma lipoprotein clearance (as specific hallmarks). The Reactome results of the top detected hits of AD genes include “Plasma lipoprotein clearance” and “Plasma lipoprotein assembly, remodeling, and clearance” and thus they were combined to more specific hallmarks as “Plasma lipoprotein assembly, remodeling, and clearance.” Similarly, all the redundant hits are combined and summarized. For more details of the categories are described (D’Eustachio, 2006; Reactome pathway knowledgebase, 2022).

We identified disease hallmarks, using the knowledgebase, GeneAnalytics (see text footnote 3) (Ben-Ari Fuchs et al., 2016) (Accessed on June 28, 2022). The knowledgebase uses a total of 74 databases. Of them, we used the results from 72 databases (Supplementary Table 1), excluding the results from 2 databases with potential reliability issues (Wikipathways and Wikipedia). The knowledgebase shows a range of *p*-values. We used the disease hits from the high tier with *p*-value ≤ 0.0001. The search provided the outcome as matched genes and the total genes, the latter of which included those genetically associated plus those differentially expressed in the database and thus it covers more genes than AD genes. Each disease was ranked based on the score obtained, which is based on (1) matched detected gene hits per total genes specific to each condition/quantitative trait locus; (2) the quality and the type of differentially expressed genes, genetic association and others; more details are described in the GeneAnalytics site above.

## Results

We updated specific biological pathways, using the latest Reactome knowledgebase analysis (Method). A total of 50 updated pathways were identified and validated with a threshold of *p*-value less than 1.00E-05 (Supplementary Table 2). Table 1 displays the top 10 hits sorted based on their *p*-value. We further eliminated the redundancies among the total 50 pathways. This process generated 11 general pathways defined as general biological hallmarks and 20 more specific pathways as defined as more specific biological hallmarks (Method). Of the 11 pathways, 5 general biological pathways are existing hallmarks reported in the earlier study (Vahdati Nia et al., 2017).

**TABLE 1:** Updated top 10 Reactome pathways.

General Reactome pathways	More specific pathways	Specific pathway	*P*-value	FDR	Reactome pathways	Symbols (HitGenes)
Metabolism of RNA	tRNA processing	tRNA processing in the mitochondrion	1.11E-16	1.05E-13	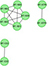	MT-TQ, MT-ND6, MT-ND4L, MT-ND4, MT-TT, MT-TR, MT-ND2, MT-ND3, MT-ND1, MT-TH, MT-CO2, MT-TG, MT-CO3, MT-TS2, MT-ATP6, MT-ATP8, MT-RNR1, MT-CYB
Transport of small molecules	Plasma lipoprotein assembly, remodeling, and clearance	Plasma lipoprotein assembly, remodeling, and clearance	3.33E-16	1.57E-13	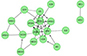	LIPA, LIPC, SOAT1, CETP, APOE, A2M, ABCA1, VLDLR, LDLR, NR1H2, ABCG1, LPL, ALB, APOA1, APOA4, APOA5, NPC1, NPC2, APOC4, APOC2, APOC1
Metabolism of RNA	rRNA processing	rRNA processing in the mitochondrion	6.00E-15	1.88E-12	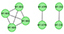	MT-ND4L, MT-ND4, MT-TT, MT-TR, MT-ND2, MT-ND3, MT-ND1, MT-TH, MT-CO2, MT-TG, MT-CO3, MT-TS2, MT-ATP6, MT-ATP8, MT-RNR1, MT-CYB
Immune system	Signaling by interleukins	Interleukin-4 and interleukin-13 signaling	1.94E-12	4.55E-10	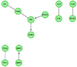	ICAM1, TP53, MAOA, PIK3R1, HMOX1, CD36, IL10, IL18, IL1A, IL1B, PTGS2, ALOX5, F13A1, TNF, TGFB1, POU2F1, IL6, IL8, MMP1, MMP3, CCL2
Transport of small molecules	Plasma lipoprotein assembly, remodeling, and clearance	Plasma lipoprotein clearance	8.06E-11	1.52E-08	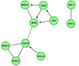	LIPA, LIPC, SOAT1, APOE, VLDLR, LDLR, NR1H2, APOA1, NPC1, NPC2, APOC4, APOC1
Immune system	Signaling by interleukins	Interleukin-10 signaling	3.56E-10	5.60E-08	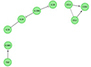	IL1RN, ICAM1, CCR2, IL10, IL18, IL1A, IL1B, PTGS2, TNF, IL6, IL8, CCL3, CCL2
Sensory perception	Visual phototransduction	Retinoid metabolism and transport	2.00E-09	2.68E-07	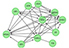	LRAT, HSPG2, APOE, LDLR, LPL, APOA1, APOA4, LRP1, LRP2, LRP8, TTR, APOC2
Metabolism of proteins	Amyloid fiber formation	Amyloid fiber formation	2.98E-09	3.48E-07	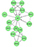	APP, HSPG2, APH1A, NCSTN, APH1B, APOE, PSENEN, BACE1, CST3, ADAM10, APOA1, APOA4, TTR, SNCA, SORL1
Transport of small molecules	Metabolism of vitamins and cofactors	Metabolism of fat-soluble vitamins	5.19E-09	5.40E-07	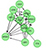	LRAT, HSPG2, APOE, LDLR, LPL, APOA1, APOA4, LRP1, LRP2, LRP8, TTR, APOC2
Immune system	Signaling by interleukins	Signaling by interleukins	1.65E-08	1.46E-06	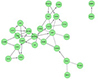	APP, IL1RN, ICAM1, MEF2A, TP53, MAOA, PIK3R1, HMOX1, CD36, CCR2, IL10, GSTO1, IL18, GAB2, IL1A, IL1B, PTGS2, ALOX5, IL33, F13A1, TNF, AGER, TGFB1, POU2F1, IL6, IL8, MMP1, MMP3, LCK, SOS2, CCL3, CCL2, S100B, SOD2

The Reactome analysis updated the biological pathways that we define as biological hallmarks (Accessed on June 28, 2022).

FDR (False detection rate). Hitgenes, full gene names and gene aliases are listed in Supplementary Table 3.

**FIGURE 1:**
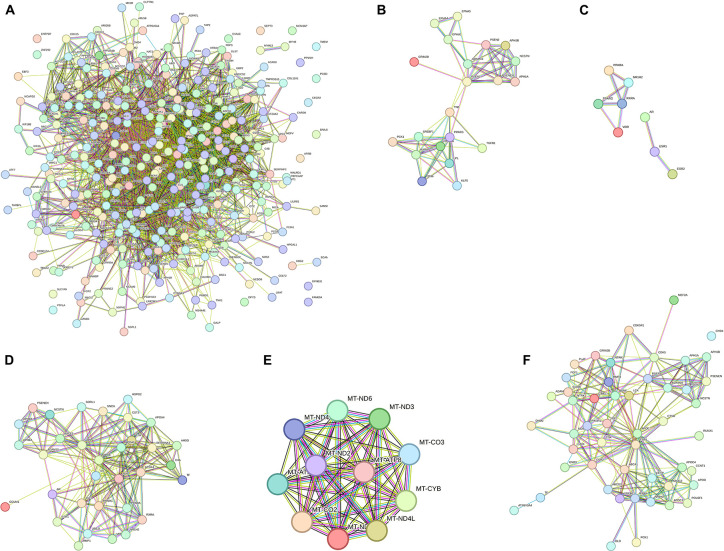
Interaction network pathways of new biological hallmarks. **(A)** Network display of all AD genes. **(B)** Developmental Biology (axon and adipose development); **(C)** gene expression (RNA polymerase II transcription); **(D)** metabolism of proteins (Amyloid formation, regulation of IGF-1/Insulin, and small ubiquitin-like modifiers/SUMOs); **(E)** metabolism of RNA (mitochondrial tRNA and rRNA processing); **(F)** signal Transduction (ErbB, NOTCH, and p75 NTR death signaling). The interaction network was created by STRING-DB. Node colors are for visual only. Edge colors are as follows: blue: from the curated database; pink: experimentally determined; black: co-expression; green: text mining.

Of the 11 pathway hallmarks, 5 were newly updated hallmarks. Figure 1 was created to display molecular interaction networks of six new hallmarks, using STRING-DB (Method). “Developmental Biology” include a subcategory in axon and adipose development. Axon development was divided into EPH-Ephrin signaling and EPH-ephrin mediated repulsion of cells; the adipose development is from transcriptional regulation of white adipocyte differentiation. “Gene expression” includes RNA Polymerase II Transcription. Although “Metabolism” is an existing hallmark, eicosanoid/steroid is a new subcategory, featuring the synthesis of 5-eicosatetraenoic acids. Notably, “Metabolism of proteins” consists of the pathway directly involved in AD (amyloid formation) and two new pathways, regulation of endocrines and lifespans (regulation of IGF-1/insulin) and protein turnover (small ubiquitin-like modifiers/SUMOs). In fact, the category of SUMOylation of intracellular receptors was significantly represented by AD genes (*p* = value of 1.19E-06; FDR of 3.82E-05) (Supplementary Table 3). Interestingly, seven genes in “SUMOylation of intracellular receptors” were shared with the genes “Gene expression (RNA polymerase II transcription)”; they were AR, PPARG, PPARA, RXRA, NR1H2, VDR, and ESR1. Another hallmark includes a new category “Metabolism of RNA,” which is mitochondrial tRNA and rRNA processing in mitochondria. Lastly, “Signal transduction” includes nuclear receptor signaling, including NR1H2 and NR1H3-mediated signaling, ErbB signaling and p75 NTR death signaling, while NOTCH signaling was reported previously (Vahdati Nia et al., 2017).

**FIGURE 2:**
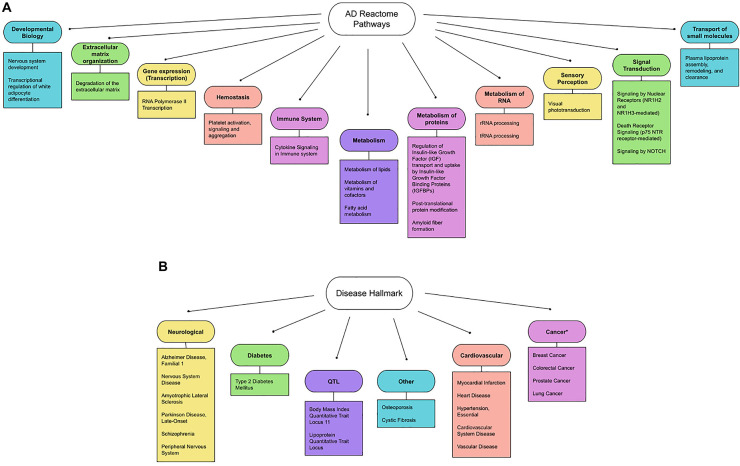
**(A)** Updated biological hallmarks. We extended the list indicated by Table 1, using the threshold (*p*-value < 1.00E-05) and summarized the 11 general biological hallmarks (circle) and more specific hallmarks (square). **(B)** Updated disease hallmarks. The diseases listed in Table 2 are organized and grouped into 6 general disease criteria. They are further classified into: (1) neurological diseases (Alzheimer’s Disease, Familial, 1, Amyotrophic Lateral Sclerosis 1, Parkinson’s Disease, Late-Onset, Schizophrenia, Peripheral Nervous System Disease, Nervous System Disease, others); (2) common age-related diseases (Diabetes, Cardiovascular Disease, Cancer and Osteoporosis); and (3) other diseases (Quantitative Trait Loci and Cystic fibrosis). Note that cancers are reported to show an inverse relationship with AD genes (indicated by asterisks).

Figure 2A summarizes 11 general pathways defined as general biological hallmarks and 20 more specific pathways (defined as more specific biological hallmarks). The 11 general hallmarks (with keywords) are in alphabetical order [asterisks (*) indicate newly identified hallmarks]:

1. Developmental Biology (axon and adipose development)*
2. Extracellular matrix organization (protein degradation)
3. Gene expression (RNA polymerase II transcription)*
4. Hemostasis (platelet regulations)
5. Immune System (interleukins)
6. Metabolism (lipoproteins, fat-soluble vitamins, eicosanoids/steroids)
7. Metabolism of proteins (Amyloid formation, regulation of IGF-1/Insulin, and small ubiquitin-like modifiers/SUMOs)*
8. Metabolism of RNA (mitochondrial tRNA and rRNA processing)*
9. Sensory perception (retinoids)
10. Signal Transduction (ErbB, NOTCH and p75 NTR death signaling)*
11. Transport of small molecules (lipoproteins)

**TABLE 2:** Diverse disease hallmarks are associated with AD genes.

General criteria	Health conditions/Loci	# Matched gene hits (total genes)*
Neurological	Alzheimer’s disease, Familial, 1	112 (836)
Neurological	Nervous system disease	91 (897)
Diabetes	Type 2 diabetes mellitus	84 (555)
Cardiovascular	Myocardial infarction	68 (302)
QTL*	Body mass index quantitative trait locus 11	76 (819)
Cancer	Breast cancer	73 (1,447)
Cardiovascular	Heart disease	61 (366)
Cardiovascular	Hypertension, essential	59 (457)
Cancer	Colorectal cancer	70 (1,492)
Neurological	Amyotrophic lateral sclerosis 1	55 (579)
Cardiovascular	Cardiovascular system disease	51 (192)
Neurological	Parkinson’s disease, late-onset	52 (387)
Other	Lipoprotein quantitative trait locus	49 (248)
Cardiovascular	Vascular disease	44 (149)
Neurological	Schizophrenia	48 (464)
Neurological	Peripheral nervous system disease	46 (369)
Cancer	Prostate cancer	56 (1,121)
Other	Osteoporosis	44 (308)
Cancer	Lung cancer	50 (1,218)
Other	Cystic fibrosis	40 (333)

The top 20 diseases in the high tier (*p*-value equal or less than 0.0001) are listed. The AD gene sets in Table 1 are used to identify diseases using all 356 AD genes (Vahdati Nia et al., 2017) and the web-based search using GeneAnalytics (Accessed on June 28, 2022).

*The number of AD gene hits (total genes classified in each group of the health conditions/Loci).

We further identified diseases associated with AD genes, using the GeneAnalytics knowledgebase (Method). The knowledgebase ranks the association based on 74 databases by tiers (Method). The detected gene hits of the top 20 diseases are summarized in Table 2 (*p*-value ≤ 0.0001). Disease hallmarks are summarized in Figure 2B. Based on the types of diseases, the AD genes can be classified as: (1) genes specific to neurological diseases; (2) genes more general to common age-related diseases; and (3) genes general to others. The first group of diseases was neurological diseases (Alzheimer’s disease, General and peripheral nervous system disease, Amyotrophic Lateral Sclerosis, Parkinson’s Disease, and Schizophrenia), which were reported previously (Vahdati Nia et al., 2017). 112 AD genes were matched out of 836 AD1 genes that are either genetically associated or differentially expressed. AD1 is a specific type of AD caused by mutations in the APP gene, a source of beta-amyloid. Of a total of 356 AD genes, the gene hits of 112 accounts for 31.4% of AD genes (112 out of 356 AD genes). The second group of diseases are common age-related diseases. They include type 2 diabetes, cardiovascular diseases (myocardial infarction, heart disease, hypertension, cardiovascular system disease, and vascular disease), cancer (breast cancer, colorectal cancer, prostate cancer, and lung cancer) and others (osteoporosis). Thus, alterations in AD genes are associated with age-related comorbidities in addition to AD. The third group introduces other conditions including cystic fibrosis and quantitative trait loci (lipoprotein and body mass index). Surprisingly, cystic fibrosis is included in the disease hit by AD genes.

## Discussion

This study updated genetic hallmarks for both biological Reactome pathways and those for diseases. We identified 11 general biological pathways, which included 5 existing pathways and 6 new pathways. The existing biological pathways include: The “immune system” unfolds pathways involving interleukin-4, 10, and 13 which are involved in the pathology of a wide variety of age-related diseases such as cardiovascular diseases, diabetes and cancers. Similarly, “Metabolism” includes lipoprotein dysregulations relevant to dyslipidemia, and cardiovascular diseases, among others. The previous version of Reactome knowledgebase classified retinoid metabolism as “Metabolism,” yet the renewed 2022 version classified it as “Sensory perception.” Retinoids or vitamin A are part of fat-soluble vitamins. Thus, we included “Sensory perception (retinoids)” back to the existing pathway of “Metabolism (fat-soluble vitamins).”

Of the 6 new biological pathways, “Metabolism of RNA” includes mitochondrial tRNA and rRNA processing in mitochondria. It may be consistent with mitochondrial deletions known to occur during aging, which may cause mitochondrial deficits (Jang et al., 2018; Swerdlow, 2018). Another category “gene expression” includes RNA Polymerase II Transcription, which is consistent with common age-related transcriptional changes (Stegeman and Weake, 2017). The category impacts the expression of a wide variety of stress response genes (Stegeman and Weake, 2017). Related to this, stress resistance is a component of life extension in model systems (Johnson et al., 2000; Murakami, 2007; Hamilton and Miller, 2016; Buono and Longo, 2018). Metabolism eicosanoid/steroid is a new subcategory including the synthesis of 5-eicosatetraenoic acids, which is a part of the eicosanoid pathways for lipoxygenase (LOX) and cyclo-oxygenase (COX) pathways among others. The category “Metabolism of proteins,” includes the pathway of amyloid formation, regulation of IGF-1/insulin, and small ubiquitin-like modifiers (SUMOs). Amyloid formation is directly involved in beta-amyloid plaques. The impaired insulin pathway causes diabetes, which is closely related to AD (Moreira, 2012; Baglietto-Vargas et al., 2016). SUMOs are involved in protein turnover which is also associated with AD (Hendriks and Vertegaal, 2016). Interestingly, SUMOylation is a post-translational modification (PTM), which controls the clearance of misfolded proteins and protein aggregations (reviewed in Vijayakumaran and Pountney, 2018). SUMOylation is involved in Alzheimer’s disease (Lee et al., 2013), Parkinson’s disease (Rott et al., 2017), Amyotrophic lateral sclerosis (Wada et al., 2020), Huntington’s disease (Sedighi et al., 2020), Prion-like proteins (Drisaldi et al., 2015), among others. Interestingly, this study identified a subcategory of “SUMOylation of intracellular receptors” including ubiquitin-conjugating enzyme E2, UBE2I (also called UBC9). The result is consistent with the finding that AD genes are associated with a wide variety of neurodegenerative diseases, while it also implies a role of receptor-mediated gene expressions controlled by SUMOylation. However, it remains unclear about the underlying molecular mechanism. Lastly, “Signal transduction” includes nuclear receptor signaling, including NR1H2 and NR1H3-mediated signaling, ErbB cancer signaling and p75 NTR death signaling; note that NOTCH signaling was reported previously (Vahdati Nia et al., 2017). NR1H2 and NR1H3 are known as liver-X receptors (LXR), which regulate cholesterol metabolism. They are receptors for their ligands, oxysterols, that are generated by the oxidation of cholesterol through ROS (reactive oxygen species) and other processes (Ma and Nelson, 2019). ErbB and p75 NTR are involved in cell survival and death.

The diseases identified by using AD genes can be categorized into 3 major criteria (neurological diseases, common age-related diseases, and others) and broken down into 6 general disease criteria. The first disease criterion is neurological diseases which include Alzheimer’s disease, general and peripheral nervous system disease, amyotrophic lateral sclerosis, Parkinson’s disease, and schizophrenia. This group has been reported and discussed in the previous study (Vahdati Nia et al., 2017). The study concluded that a single gene alteration may cause multiple neurological diseases. The top hit of the disease in this study was AD1 (Alzheimer’s Disease, Familial, 1). Based on affected genes, the efforts on classifying AD have been ongoing and currently classified from AD1 to AD16. AD1 is caused by mutations in the APP genes. AD2 is associated with the ApoE4 allele. AD3 is caused by mutations in PSEN1. AD4 is caused by mutations in the PSEN2 gene. For more details, see the GTR (genetic testing registry) at the NCBI (National Center for Biotechnology Information database) [Genetic Testing Registry [GTR], 2022, Accessed July 12, 2022]. Due to the number of AD genes, the list of AD types is expected to be increased in number. The study provides a clear example of genetic and phenotypic heterogeneity. The result is consistent with the complex clinical presentations (i.e., clinical heterogeneity) of AD (Ferrari and Sorbi, 2021).

The second and third disease criteria include common age-related diseases. Age-related diseases are also seen in people as age-related comorbidities, with which two or more diseases commonly occur in a single person. The comorbidities include diabetes (type 2 diabetes), cardiovascular diseases (myocardial infarction, heart disease, hypertension, cardiovascular system disease, and vascular disease), cancer (breast cancer, colorectal cancer, prostate cancer, and lung cancer) and others (osteoporosis). It is worth noting that cancers show an inverse relation with AD (Nudelman et al., 2019). The observation is consistent with the previous studies that a major hypertension target, angiotensin-converting enzyme (ACE) is also involved in AD (Le et al., 2020, 2021). Despite being less defined, AD may be classified as type 3 diabetes, which is a type of diabetes in the brain (Steen et al., 2005; Pilcher, 2006; de la Monte, 2014; Leszek et al., 2017). The vast majority of AD falls into LOAD, whose onset occurs starting at 65 years of age, while age-related diseases occur earlier than that. Although age-related comorbidities are known to be vulnerable to a variety of conditions, for example, COVID-19 (Antos et al., 2021), the straightforward interpretation of the result is that AD genes are associated with AD as well as with common age-related diseases.

It is conceptually important that the AD genes define AD as a part of age-related comorbidities with shared biological mechanisms. While the study raises the possibility that age-related diseases may lead to AD, we are more inclined to the possibility that the shared biological mechanisms may lead to AD and other age-related comorbidities. We are beginning to learn that “there is growing evidence that people who adopt healthy lifestyle habits…can lower their risk of dementia…which have been shown to prevent cancer, diabetes, and heart disease (Centers for Disease Control and Prevention [CDC], 2020).” Moreover, the CDC describes the broad neurological behavioral warning signs of Alzheimer’s disease, such as memory impairment, difficulty in daily tasks, and poor judgment, among others [Hamilton and Miller, 2016]. This study further suggests that common age-related comorbidities may present early signs when AD genetics is involved. Related to this, a wide variety of clinical scenarios may be considered. For example, people living with AD gene alterations may have common age-related comorbidities and risk of AD development; people living with AD gene alterations may have AD with other common age-related comorbidities or people living with AD gene alterations may develop neurological and other conditions.

The cure for AD is still unknown. Currently, Aducanumab, a human anti-beta-amyloid antibody, is the only disease-modifying medication approved by FDA (U.S. Food and Drug Administration) (National Institute on Aging, 2021; Alzheimer’s Association, 2022). The medication requires assessment of brain beta-amyloid, which uses PET (positron emission tomography) scans or analysis of cerebrospinal fluid. As a clinical approach to AD, we present that age-related comorbidities may provide an early assessment when genetic testing is performed. We also present that the treatment options for age-related comorbidities may be effective when biological mechanisms are considered. Alternatively, the implications from the model systems may be useful for treatment options. Stress resistance confers resistance to multiple forms of stressors, such as pathogens and the toxic beta-amyloid, which is tightly associated with Alzheimer’s disease in the model systems (Florez-McClure et al., 2007; Machino et al., 2014). Multiplex stress resistance is a key to understanding the mechanism of extended lifespans and health spans (Murakami et al., 2003; Murakami, 2006). Additionally, stress resistance is tightly associated with life-extending interventions (Murakami, 2006) in which the molecular mechanisms are genetically characterized, for example, the insulin/IGF-1 pathways (Murphy and Hu, 2013), and serotonin pathways (Murakami and Murakami, 2007), among others; these can be assessed by semi-automated systems (Machino et al., 2014). The IGF-1/insulin pathways are a major regulator of lifespans (Finch and Ruvkun, 2001; Kenyon, 2010; Ewald et al., 2018; Zhang et al., 2020) and are involved in age-related memory impairment (Murakami et al., 2005). Similarly, the serotonin pathways regulate age-related behavioral changes, lifespans and stress resistance (Murakami and Murakami, 2007; Murakami et al., 2008). More details of age-related memory impairment and a related theory (middle-life crisis theory of aging) are described elsewhere (Murakami et al., 2011; Murakami, 2013). There is an increasing number of the study using meta-analysis and GWAS (genome-wide association studies) (e.g., Jansen et al., 2019; Kunkle et al., 2019; Wightman et al., 2021; Bellenguez et al., 2022) and proteomics (Bai et al., 2020), which confirmed our earlier study (Vahdati Nia et al., 2017). This study provides an updated view of genetic and disease hallmarks. Taken together, we suggest that this study of revisiting AD genes provides the strength of treatment options as well as future direction. It will be a powerful way to develop a science-based tool for the long-waited diagnosis, prevention, and treatment options for AD.

## Data availability statement

The original contributions presented in this study are included in the article/Supplementary material, further inquiries can be directed to the corresponding author.

## Author contributions

SM was involved in all aspects of this research. PL was involved in data analysis and presentation. Both authors contributed to the article and approved the submitted version.

## Footnotes

1
http://alzgene.org/

2
http://reactome.org/

3
http://geneanalytics.genecards.org/

4
http://string-db.org/

## References

[B1] Alzheimer’s Association (2022). *Aducanumab approved for treatment of Alzheimer’s disease.* Chicago, IL: Alzheimer’s Association.

[B2] Antos A. Kwong M. L. Balmorez T. Villanueva A. Murakami S. (2021). Unusually high risks of COVID-19 mortality with age-related comorbidities: An adjusted meta-analysis method to improve the risk assessment of mortality using the comorbid mortality data. *Infect. Dis. Rep.* 13 700–711. 10.3390/idr13030065 34449622 PMC8395741

[B3] Baglietto-Vargas D. Shi J. Yaeger D. M. Ager R. LaFerla F. M. (2016). Diabetes and Alzheimer’s disease crosstalk. *Neurosci. Biobehav. Rev.* 64 272–287. 10.1016/j.neubiorev.2016.03.005 26969101

[B4] Bai B. Wang X. Li Y. Chen P. C. Yu K. Dey K. K. (2020). Deep multilayer brain proteomics identifies molecular networks in Alzheimer’s disease progression. *Neuron* 105 975–991.e7. 10.1016/j.neuron.2019.12.015 31926610 PMC7318843

[B5] Bateman R. J. Xiong C. Benzinger T. L. Fagan A. M. Goate A. Fox N. C. (2012). Dominantly inherited Alzheimer network. Clinical and biomarker changes in dominantly inherited Alzheimer’s disease. *N. Engl. J. Med.* 367 795–804. 10.1056/NEJMoa1202753 22784036 PMC3474597

[B6] Bellenguez C. Küçükali F. Jansen I. E. Kleineidam L. Moreno-Grau S. Amin N. (2022). New insights into the genetic etiology of Alzheimer’s disease and related dementias. *Nat. Genet.* 54 412–436. 10.1038/s41588-022-01024-z 35379992 PMC9005347

[B7] Ben-Ari Fuchs S. Lieder I. Stelzer G. Mazor Y. Buzhor E. Kaplan S. (2016). GeneAnalytics: An integrative gene set analysis tool for next generation sequencing, RNAseq and microarray data. *OMICS* 20 139–151. 10.1089/omi.2015.0168 26983021 PMC4799705

[B8] Bertram L. McQueen M. B. Mullin K. Blacker D. Tanzi R. E. (2007). Systematic meta-analyses of Alzheimer disease genetic association studies: The AlzGene database. *Nat. Genet.* 39 17–23. 10.1038/ng1934 17192785

[B9] Buono R. Longo V. D. (2018). Starvation, stress resistance, and cancer. *Trends Endocrinol. Metab.* 29 271–280. 10.1016/j.tem.2018.01.008 29463451 PMC7477630

[B10] Centers for Disease Control and Prevention [CDC] (2020). *Alzheimer’s disease and related dementias.* Atlanta, GA: Centers for Disease Control and Prevention.

[B11] D’Eustachio P. (2006). Reactome – a curated knowledgebase of biological pathways. *Lipoprotein Metab.* 17. 10.3180/react_6823.1

[B12] de la Monte S. M. (2014). Type 3 diabetes is sporadic Alzheimer’s disease: Mini-review. *Eur. Neuropsychopharmacol.* 24 1954–1960. 10.1016/j.euroneuro.2014.06.008 25088942 PMC4444430

[B13] Drisaldi B. Colnaghi L. Fioriti L. Rao N. Myers C. Snyder A. M. (2015). SUMOylation is an inhibitory constraint that regulates the prion-like aggregation and activity of CPEB3. *Cell Rep.* 11 1694–1702. 10.1016/j.celrep.2015.04.061 26074071 PMC5477225

[B14] Ewald C. Y. Castillo-Quan J. I. Blackwell T. K. (2018). Untangling longevity, Dauer, and Healthspan in *Caenorhabditis elegans* insulin/IGF-1-signalling. *Gerontology* 64 96–104. 10.1159/000480504 28934747 PMC5828946

[B15] Ferrari C. Sorbi S. (2021). The complexity of Alzheimer’s disease: An evolving puzzle. *Physiol. Rev.* 101 1047–1081. 10.1152/physrev.00015.2020 33475022

[B16] Finch C. E. Ruvkun G. (2001). The genetics of aging. *Annu. Rev. Genomics Hum. Genet.* 2 435–462. 10.1146/annurev.genom.2.1.435 11701657

[B17] Florez-McClure M. L. Hohsfield L. A. Fonte G. Bealor M. T. Link C. D. (2007). Decreased insulin-receptor signaling promotes the autophagic degradation of beta-amyloid peptide in *Caenorhabditis elegans*. *Autophagy* 3 569–580. 10.4161/auto.4776 17675890

[B18] Genetic Testing Registry [GTR], (2022). National center for biotechnology information. *Alzheimer Dis.*

[B19] Gillespie M. Jassal B. Stephan R. Milacic M. Rothfels K. Senff-Ribeiro A. (2022). The reactome pathway knowledgebase 2022. *Nucleic Acids Res.* 50 D687–D692. 10.1093/nar/gkab1028 34788843 PMC8689983

[B20] Hamilton K. L. Miller B. F. (2016). What is the evidence for stress resistance and slowed aging? *Exp. Gerontol.* 82 67–72. 10.1016/j.exger.2016.06.001 27268049

[B21] Hendriks I. A. Vertegaal A. C. (2016). A comprehensive compilation of SUMO proteomics. *Nat. Rev. Mol. Cell Biol.* 17 581–595. 10.1038/nrm.2016.81 27435506

[B22] Jang J. Y. Blum A. Liu J. Finkel T. (2018). The role of mitochondria in aging. *J. Clin. Invest.* 128 3662–3670. 10.1172/JCI120842 30059016 PMC6118639

[B23] Jansen I. E. Savage J. E. Watanabe K. Bryois J. Williams D. M. Steinberg S. (2019). Genome-wide meta-analysis identifies new loci and functional pathways influencing Alzheimer’s disease risk. *Nat. Genet.* 51 404–413. 10.1038/s41588-018-0311-9 30617256 PMC6836675

[B24] Johnson T. E. Cypser J. de Castro E. de Castro S. Henderson S. Murakami S. (2000). Gerontogenes mediate health and longevity in nematodes through increasing resistance to environmental toxins and stressors. *Exp. Gerontol.* 35 687–694. 10.1016/s0531-5565(00)00138-8 11053658

[B25] Kenyon C. J. (2010). The genetics of ageing. *Nature* 464 504–512. 10.1038/nature08980 20336132

[B26] Kunkle B. W. Grenier-Boley B. Sims R. Bis J. C. Damotte V. Naj A. C. (2019). Genetic meta-analysis of diagnosed Alzheimer’s disease identifies new risk loci and implicates Aβ, tau, immunity and lipid processing. *Nat. Genet.* 51 414–430. 10.1038/s41588-019-0358-2 30820047 PMC6463297

[B27] Le D. Brown L. Malik K. Murakami S. (2021). Two opposing functions of angiotensin-converting enzyme (ACE) that links hypertension, dementia, and aging. *Int. J. Mol. Sci.* 22:13178. 10.3390/ijms222413178 34947975 PMC8707689

[B28] Le D. Crouch N. Villanueva A. Ta P. Dmitriyev R. Tunzi M. (2020). Evidence-based genetics and identification of key human Alzheimer’s disease alleles with co-morbidities. *J. Neurol. Exp. Neurosci.* 6 20–24. 10.17756/jnen.2020-069

[B29] Lee L. Sakurai M. Matsuzaki S. Arancio O. Fraser P. (2013). SUMO and Alzheimer’s disease. *Neuromolecular Med.* 15 720–736. 10.1007/s12017-013-8257-7 23979993 PMC3823823

[B30] Leszek J. Trypka E. Tarasov V. V. Ashraf G. M. Aliev G. (2017). Type 3 diabetes mellitus: A novel implication of Alzheimers disease. *Curr. Top. Med. Chem.* 17 1331–1335. 10.2174/1568026617666170103163403 28049395

[B31] Ma L. Nelson E. R. (2019). Oxysterols and nuclear receptors. *Mol. Cell. Endocrinol.* 484 42–51. 10.1016/j.mce.2019.01.016 30660701

[B32] Machino K. Link C. D. Wang S. Murakami H. Murakami S. (2014). A semi-automated motion-tracking analysis of locomotion speed in the *Caenorhabditis elegans* transgenics overexpressing beta-amyloid in neurons. *Front. Genet.* 5:202. 10.3389/fgene.2014.00202 25071831 PMC4082091

[B33] Matthews K. A. Xu W. Gaglioti A. H. Holt J. B. Croft J. B. Mack D. (2018). Racial and ethnic estimates of Alzheimer’s disease and related dementias in the United States (2015–2060) in adults aged ≥ 65 years. *Alzheimers Dement.* 15 17–24. 10.1016/j.jalz.2018.06.3063 30243772 PMC6333531

[B34] Moreira P. I. (2012). Alzheimer’s disease and diabetes: An integrative view of the role of mitochondria, oxidative stress, and insulin. *J Alzheimers Dis.* 30 Suppl 2 S199–S215. 10.3233/JAD-2011-111127 22269163

[B35] Murakami H. Murakami S. (2007). Serotonin receptors antagonistically modulate *Caenorhabditis elegans* longevity. *Aging Cell* 6 483–488. 10.1111/j.1474-9726.2007.00303.x 17559503

[B36] Murakami H. Bessinger K. Hellmann J. Murakami S. (2005). Aging-dependent and -independent modulation of associative learning behavior by insulin/insulin-like growth factor-1 signal in *Caenorhabditis elegans*. *J. Neurosci.* 25 10894–10904. 10.1523/JNEUROSCI.3600-04.2005 16306402 PMC6725869

[B37] Murakami H. Bessinger K. Hellmann J. Murakami S. (2008). Manipulation of serotonin signal suppresses early phase of behavioral aging in *Caenorhabditis elegans*. *Neurobiol. Aging* 29 1093–1100. 10.1016/j.neurobiolaging.2007.01.013 17336425

[B38] Murakami S. (2006). Stress resistance in long-lived mouse models. *Exp. Gerontol.* 41 1014–1019. 10.1016/j.exger.2006.06.061 16962277

[B39] Murakami S. (2007). *Caenorhabditis elegans* as a model system to study aging of learning and memory. *Mol. Neurobiol.* 35 85–94. 10.1007/BF02700625 17519507

[B40] Murakami S. (2013). “Age-dependent modulation of learning and memory in *Caenorhabditis elegans*,” in *Invertebrate learning and memory; handbook of behavioral neuroscience*, eds Menzel R. Benjamin P. R. (Cambridge, MA: Elsevier/Academic Press), 140–150.

[B41] Murakami S. Cabana K. Anderson D. (2011). “Current advances in the study of oxidative stress and age-related memory impairment in *Caenorhabditis elegans*,” in *Molecular aspects of oxidative stress on cell signaling in vertebrates and invertebrates*, eds Farooqui T. Farooqui A. (Hoboken, NJ: John Wiley & Sons), 347–360.

[B42] Murakami S. Salmon A. Miller R. A. (2003). Multiplex stress resistance in cells from long-lived dwarf mice. *FASEB J.* 17 1565–1566. 10.1096/fj.02-1092fje 12824282

[B43] Murphy C. T. Hu P. J. (2013). Insulin/insulin-like growth factor signaling in *Caenorhabditis elegans*. *WormBook* 26 1–43. 10.1895/wormbook.1.164.1 24395814 PMC4780952

[B44] National Institute on Aging (2021). *How is Alzheimer’s disease treated?.* Bethesda, MD: National Institute on Aging.

[B45] Nudelman K. N. H. McDonald B. C. Lahiri D. K. Saykin A. J. (2019). Biological hallmarks of cancer in Alzheimer’s disease. *Mol. Neurobiol.* 56 7173–7187. 10.1007/s12035-019-1591-5 30993533 PMC6728183

[B46] Pilcher H. (2006). Alzheimer’s disease could be “type 3 diabetes”. *Lancet Neurol.* 5 388–389. 10.1016/s1474-4422(06)70434-3 16639835

[B47] Rott R. Szargel R. Shani V. Hamza H. Savyon M. Abd Elghani F. (2017). SUMOylation and ubiquitination reciprocally regulate α-synuclein degradation and pathological aggregation. *Proc. Natl. Acad. Sci. U.S.A.* 114 13176–13181. 10.1073/pnas.1704351114 29180403 PMC5740625

[B48] Sedighi F. Adegbuyiro A. Legleiter J. (2020). SUMOylation prevents huntingtin fibrillization and localization onto lipid membranes. *ACS Chem. Neurosci.* 11 328–343. 10.1021/acschemneuro.9b00509 31880908

[B49] Sherva R. Kowall N. W. (2022). “Genetics of Alzheimer disease,” in *UpToDate*, eds Steven T. DeKosky S. T. Raby B. A. Wilterdink J. L. (Waltham, MA: UpToDate).

[B50] Steen E. Terry B. M. Rivera E. J. Cannon J. L. Neely T. R. Tavares R. (2005). Impaired insulin and insulin-like growth factor expression and signaling mechanisms in Alzheimer’s disease–is this type 3 diabetes? *J. Alzheimers Dis.* 7 63–80. 10.3233/jad-2005-7107 15750215

[B51] Stegeman R. Weake V. M. (2017). Transcriptional signatures of aging. *J. Mol. Biol.* 429 2427–2437. 10.1016/j.jmb.2017.06.019 28684248 PMC5662117

[B52] Swerdlow R. H. (2018). Mitochondria and mitochondrial cascades in Alzheimer’s disease. *J. Alzheimers Dis.* 62 1403–1416. 10.3233/JAD-170585 29036828 PMC5869994

[B53] Szklarczyk D. Morris J. H. Cook H. Kuhn M. Wyder S. Simonovic M. (2017). The STRING database in 2017: Quality-controlled protein-protein association networks, made broadly accessible. *Nucleic Acids Res.* 45 D362–D368. 10.1093/nar/gkw937 27924014 PMC5210637

[B54] Vahdati Nia B. Kang C. Tran M. G. Lee D. Murakami S. (2017). Meta analysis of human AlzGene database: Benefits and limitations of using *Caenorhabditis elegans* for the study of Alzheimer’s disease and co-morbid conditions. *Front. Genet.* 8:55. 10.3389/fgene.2017.00055 28553317 PMC5427079

[B55] Vaz M. Silvestre S. (2020). Alzheimer’s disease: Recent treatment strategies. *Eur. J. Pharmacol.* 887:173554. 10.1016/j.ejphar.2020.173554 32941929

[B56] Vijayakumaran S. Pountney D. L. (2018). SUMOylation, aging and autophagy in neurodegeneration. *Neurotoxicology* 66 53–57. 10.1016/j.neuro.2018.02.015 29490232

[B57] Wada H. Suzuki D. Niikura T. (2020). Regulation of ALS-associated SOD1 mutant SUMOylation and aggregation by SENP and PIAS family proteins. *J. Mol. Neurosci.* 70 2007–2014. 10.1007/s12031-020-01604-w 32462635

[B58] Wightman D. P. Jansen I. E. Savage J. E. Shadrin A. A. Bahrami S. Holland D. (2021). A genome-wide association study with 1,126,563 individuals identifies new risk loci for Alzheimer’s disease. *Nat. Genet.* 53 1276–1282. 10.1038/s41588-021-00921-z 34493870 PMC10243600

[B59] Zhang Z. D. Milman S. Lin J. R. Wierbowski S. Yu H. Barzilai N. (2020). Genetics of extreme human longevity to guide drug discovery for healthy ageing. *Nat. Metab.* 2 663–672. 10.1038/s42255-020-0247-0 32719537 PMC7912776

